# Structural design of highly permeable Bi_2_O_3_ microspheres decorated by Pt-nanoparticles: facile synthesis and acetic acid sensing performance

**DOI:** 10.1007/s12598-025-03391-y

**Published:** 2025-07-10

**Authors:** Fan Yang, Jun-Ning Zhang, Chao Zhang, Xin-Da Xu, Bing Li, Woochul Yang, Wan-Feng Xie

**Affiliations:** 1 College of Electronics and Information, Qingdao University, Qingdao, 266071 China; 2 Department of Physics, Dongguk University, Seoul, 04620 Republic of Korea; 3 Institute for Materials Discovery, Department of Chemistry, University College London, London, WC1E 7JE UK

**Keywords:** Semiconducting metal oxide, Bi_2_O_3_ microspheres, Acetic acid, Gas sensor

## Abstract

Real-time detection of acetic acid vapor is of concern for ensuring environmental and personal safety. However, acetic acid gas sensors, particularly those based on Bi_2_O_3_, often fail to meet practical performance requirements owing to their slow response characteristics and high operating temperature. To enhance sensing performance, highly permeable Bi_2_O_3_ microspheres decorated by Pt-nanoparticles are rationally synthesized by a facile template method. Among the fabricated sensors, the one based on 3 wt% Pt-decorated Bi_2_O_3_ demonstrated excellent sensing performance. Specifically, the sensor displayed high selectivity for acetic acid, rapid response and recovery times (22.5 and 9 s, respectively), strong resistance to interference, and good long-term stability at a low operating temperature (150 °C). Notably, the sensor exhibited an exceptionally high response of 126 to 100 ppm acetic acid—the highest reported value for Bi_2_O_3_-based sensors tested at a relatively low operating temperature in recent years. These results demonstrate that Pt-decorated Bi_2_O_3_ holds strong potential for use in high-performance acetic acid sensors.

**Graphical abstract:**

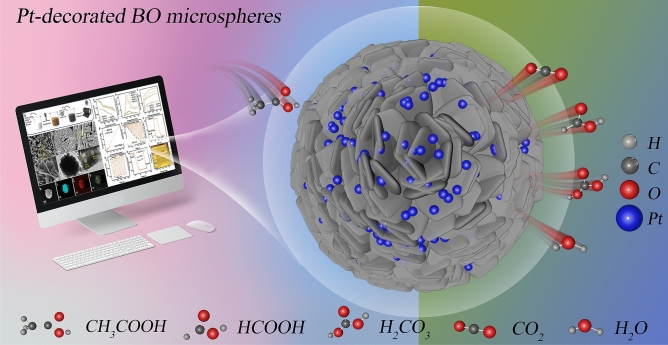

**Supplementary Information:**

The online version contains supplementary material available at 10.1007/s12598-025-03391-y.

## 中文摘要

乙酸的实时检测对保护环境和人身安全具有重要意义。然而，乙酸气体传感器，特别是基于Bi_2_O_3_的传感器，由于其响应慢，工作温度高，不能满足实际应用的性能要求。为了提高其传感性能，采用简单模板法合理合成了由Pt纳米粒子修饰的高穿透Bi_2_O_3_微球。在所制备的传感器中，基于3 wt% Pt修饰Bi_2_O_3_的传感器表现出优异的传感性能。该传感器对乙酸的选择性好，响应时间和恢复时间快（22.5 s和9 s），抗干扰能力强，长期稳定和工作温度低（150 °C）。值得注意的是，该传感器对100 ppm的乙酸表现出超高的响应（126），这是近年来，在相对较低的工作温度下Bi_2_O_3_基乙酸传感器达到的最高响应。结果表明，Pt修饰的Bi_2_O_3_在高性能乙酸传感器中具有很强的应用潜力。

## Introduction

Acetic acid, one of the most important organic acids, is a bulk chemical widely used in industry. While it can promote metabolism in small quantities, excessive ingestion or prolonged exposure may be harmful to human health. It is also a strong irritant and may cause skin inflammation and allergic reactions upon prolonged contact [1]. Ingesting large amounts of acetic acid can lead to gastrointestinal discomfort and, in severe cases, may result in gastritis, gastric ulcers, and other related conditions [2]. Thus, to ensure industrial production safety and safeguard human health, detecting acetic acid in complex environments is essential. However, the complexity and high cost of conventional detection methods limit their widespread application [3].

To date, various types of gas sensors have been developed, including electrochemical, catalytic combustion, infrared, and thermal conductivity sensors. However, rising living standards and the growing concern for environmental protection have increased the demand for high-performance gas sensors capable of effectively monitoring air pollution, industrial emissions, and food and indoor environment quality. Among these, chemically resistant gas sensors based on metal oxide semiconductors (MOSs)—such as Bi_2_O_3_ [4], WO_3_ [5], ZnO [6], SnO_2_ [7], In_2_O_3_ [8]—have been widely used for detecting toxic, combustible, and explosive gases owing to their ease of fabrication, low cost, compact size, and other favorable characteristics. Because many toxic and combustible gases are colorless, odorless, tasteless, and hazardous even at low concentrations, gas sensors based on MOSs must exhibit rapid response characteristics, high sensitivity, and strong selectivity toward trace levels of target gases [9]. Given that, the gas sensing mechanism of gas sensors strongly depends on surface reactions between target gases and chemisorbed oxygen species on the active surface [10], strategies such as the controlled synthesis of highly active nanomaterials, tailored design of sensor structures [11], and integration of effective catalytic sensitization are viable for developing high-performance gas sensors [12].

In a previous study, we synthesized highly permeable Bi_2_O_2_CO_3_ microspheres through nanostructural control and verified that their morphology is well-suited for gas sensor applications [13]. However, their poor thermal stability resulted in unstable sensing performance at elevated temperatures. Bi_2_O_3_ has been widely reported as an excellent sensing material owing to its unique physicochemical properties [14]. For instance, a Bi_2_O_3_-nanoplate-based sensor has been reported to demonstrate a 100% gas response at a low operating temperature of 150 °C and to offer a detection limit of up to 11,000 ppm for CO [15]. Similarly, an Ag/Bi_2_O_3_-nanocomposite-based sensor has been documented to exhibit a gas response of 89.2% to toluene vapor (50 ppm) at room temperature [16]. These findings suggest that a novel permeable Bi_2_O_3_ nanostructure derived from Bi_2_O_2_CO_3_ could serve as a promising sensing material. Therefore, in this study, we convert Bi_2_O_2_CO_3_ microspheres into thermally stable Bi_2_O_3_ through calcination in air while preserving their highly permeable structure.

Although converting Bi_2_O_2_CO_3_ to Bi_2_O_3_ improves the thermal stability of the final product, experimental results reveal that the sensor response still requires enhancement. The performance of MOS-based gas sensors primarily depends on redox reactions occurring on the surface of the sensing material. Extensive theoretical and experimental studies have demonstrated that noble metal decoration—such as Pt decoration—plays a crucial role in enhancing the surface reactivity of MOS materials [5]. For instance, a previous study reported that a Pt-nanoparticle-functionalized sensing material exhibited a 31.4-fold improvement in its NH_3_ sensing response, rising from 6.48 to 203.44 at 500 ppm. This dramatic improvement confirms the exceptional sensitivity of Pt-decorated SnO_2_ sensors [17]. Another study reported that Pt-decorated ZnO polyhedra exhibited a 9% response to 500 ppb CO [18], primarily attributed to the catalytic and electron sensitization effects of Pt decoration. These results highlight the value of Pt decoration in enhancing sensing performance.

In this study, a high-performance acetic acid sensor was fabricated by combining highly permeable Bi_2_O_2_CO_3_ microspheres with Pt, a noble metal catalyst. As anticipated, the resulting Pt-decorated Bi_2_O_3_ microspheres displayed a highly permeable layered spherical structure. The microspheres exhibited an average diameter of 2.4 μm, and the average thickness of most layers was approximately 170 nm. Consequently, the sensor based on 3 wt% Pt-decorated Bi_2_O_3_ exhibited a strong response toward acetic acid (126) and a rapid recovery time of 9 s at a low operating temperature of 150 °C (100 ppm), along with excellent interference resistance. These findings demonstrate the feasibility of using Bi_2_O_3_-Pt microspheres for acetic acid detection and provide a promising route for the development of high-performance gas sensors.

## Experimental

### Preparation of the Bi_2_O_3_ precursor

All chemicals were obtained from Aladdin. First, the Bi_2_O_3_ precursor was prepared using a hydrothermal method. Briefly, 1 mmol bismuth nitrate pentahydrate (Bi(NO_3_)_3_·5H_2_O) was evenly dispersed in 35 mL deionized (DI) water. Subsequently, 2 mmol urea (CH_4_N_2_O) and 2 mmol trisodium citrate (C_6_H_5_Na_3_O_7_·2H_2_O) were added to the mixture. After magnetic stirring for 60 min, a uniform solution was obtained. The resulting mixture was transferred to a 50 mL Teflon-lined stainless-steel autoclave and maintained at 160 °C for 24 h. The resulting product was washed five times with DI water and anhydrous ethanol, following which it was dried in an oven at 60 °C for 6 h. The Bi_2_O_3_ precursor was finally obtained through calcination in a tubular furnace at 400 °C for 2 h with a heating rate of 1 °C min^−1^. The resulting product was designated as Bi_2_O_3_-Pt-0.

### Preparation of the Pt-decorated Bi_2_O_3_ sensing materials

The sensing materials (Bi_2_O_3_-Pt) were synthesized using the sodium borohydride (NaBH_4_) reduction method. First, 100 mg of the synthesized precursor was dispersed evenly in 35 mL of DI water. Subsequently, varying amounts of H_2_PtCl_6_ (1, 2, 3 or 4 mg) were added to the mixture, followed by ultrasonication for 5 min and magnetic stirring for 30 min. An NaBH_4_ solution (2 mL, 0.1 mol L^−1^) was then slowly added under continuous stirring for an additional 30 min. The resulting suspension was washed and dried as previously described. For convenience, the products were designated as Bi_2_O_3_-Pt-1, Bi_2_O_3_-Pt-2, Bi_2_O_3_-Pt-3, and Bi_2_O_3_-Pt-4, based on the amount of added H_2_PtCl_6_.

### Characterization of sensing materials

X-ray diffraction (XRD) patterns were acquired on a Rigaku Ultima IV diffractometer (Cu Kα, *λ* = 0.15418 nm) at a scanning rate of 1° min^−1^. The morphology of the microspheres was examined using field-emission scanning electron microscopy (FE-SEM, Zeiss Gemini 500) and transmission electron microscopy (TEM, FEI Talos F200x). The valence states of the elements were determined using X-ray photoelectron spectroscopy (XPS, Thermo Fisher ESCALAB XI +).

### Sensor fabrication and gas sensing measurements

First, each sensing material was mixed with anhydrous ethanol and thoroughly ground to form a uniform paste. This paste was then carefully drop-coated onto a ceramic tube. Next, a small Ni–Cr alloy coil was embedded within the ceramic tube for operating temperature regulation, as illustrated in Fig. S1. Gas sensing measurements were conducted using a WS-30A gas sensing system at approximately 60% relative humidity (RH). During the sensing performance test, a specified amount of the target liquid was injected into the evaporation platform (18 L) of the test chamber. The purity of each target gas component is listed in Table S1. The liquid was heated, vaporized, and evenly dispersed throughout the test chamber. Once the sensor resistance stabilized, the chamber was opened to allow ambient air to enter. The concentration of the target gas (*C*) was calculated using Eq. (1):

1 $$\begin{array}{c}{V}_{\text{x}}=\frac{V\times C\times M}{22.4\times D\times P}\times \frac{273.15+{T}_{\text{R}}}{273.15+{T}_{\text{B}}}\times {10}^{-9}\end{array}$$

where *V*_x_ denotes the volume of the liquid reagent (mL), *V* represents the volume of the test chamber (18 L), *C* denotes the concentration of the target gas (ppm), *M* represents the molecular weight of the reagent (g mol^−1^), *D* denotes the density of the liquid reagent (g mL^−1^, 20 °C), *P* represents the purity of the liquid reagent (%), *T*_R_ signifies room temperature (25 °C), and *T*_B_ denotes the experimental temperature (°C).

## Results and discussion

**Fig. 1:**
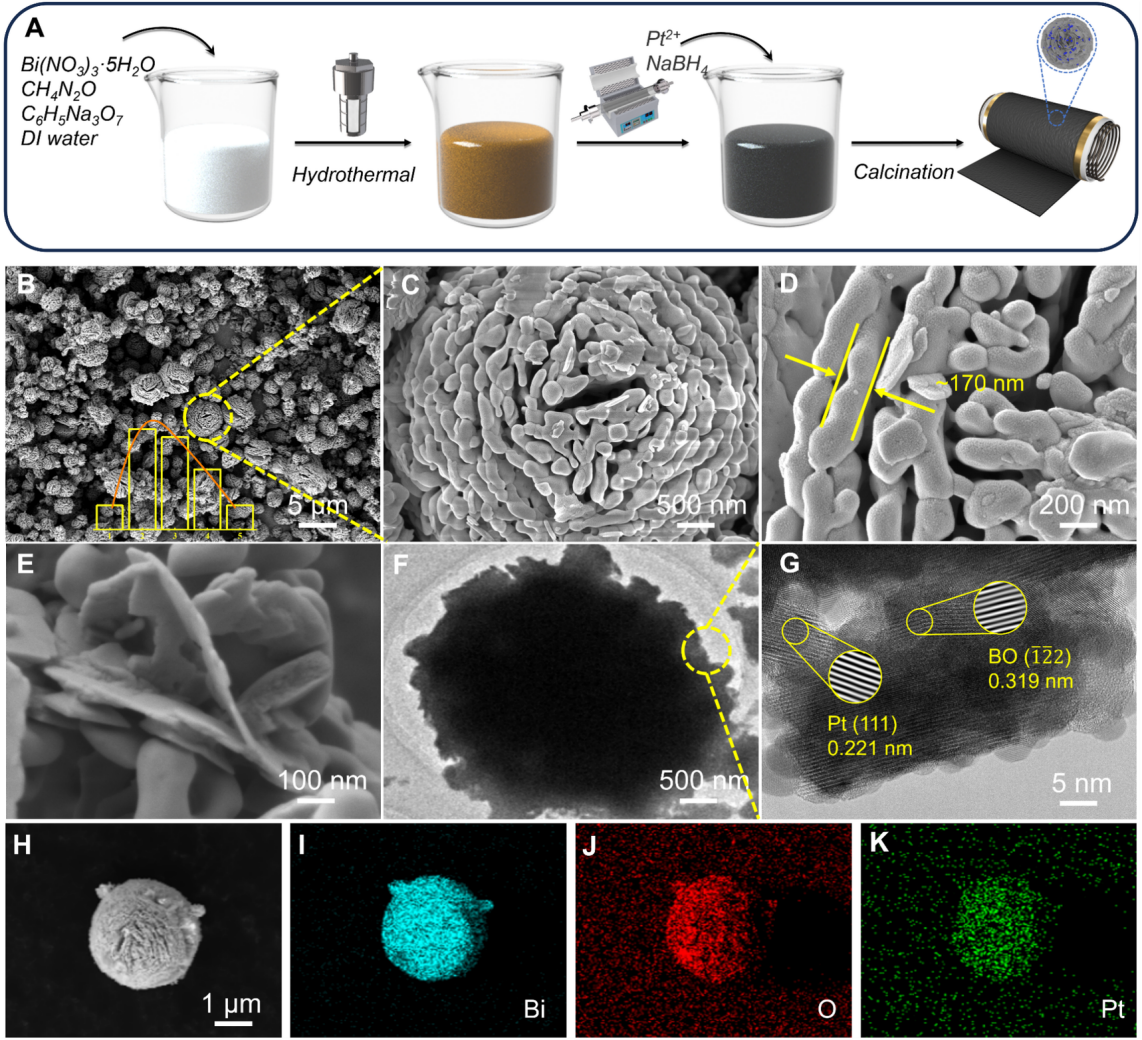
**A** Schematic illustration of the synthesis of the Pt-modified Bi_2_O_3_ and fabrication of gas sensor; **B**–**E** SEM images of the Pt-decorated Bi_2_O_3_; **F**, **G** TEM images of the Pt-decorated Bi_2_O_3_ with different resolutions; **H**–**K** EDS elemental mapping of the Pt-decorated Bi_2_O_3_

Bi_2_O_3_-Pt microspheres were synthesized using a facile hydrothermal method followed by NaBH_4_ reduction, as depicted in Fig. 1A. The successful synthesis of Pt-decorated Bi_2_O_3_ microspheres was confirmed by SEM. The data in Fig. 1B reveal an average microsphere diameter of 2.4 μm. High-magnification images (Fig. 1C, D) indicate that each microsphere features a layered, porous structure with an average layer thickness of approximately 170 nm. Figure 1E indicates that each layer consists of multilayered sheets, resulting in a slightly greater thickness compared to that reported in a previous study [13]. TEM images further confirm the microsphere structure of Pt-decorated Bi_2_O_3_ (Fig. 1F). High-resolution TEM (HR-TEM) images (Fig. 1G) reveal lattice spacings of 0.221 and 0.319 nm, corresponding to the (111) crystal faces of Pt and the ($\overline{1 }\overline{2}2)$ faces of Bi_2_O_3_. Energy-dispersive X-ray spectroscopy (EDS) analysis confirms that Bi, Pt, and O are evenly distributed throughout the Bi_2_O_3_ microspheres (Fig. 1H–K).

**Fig. 2:**
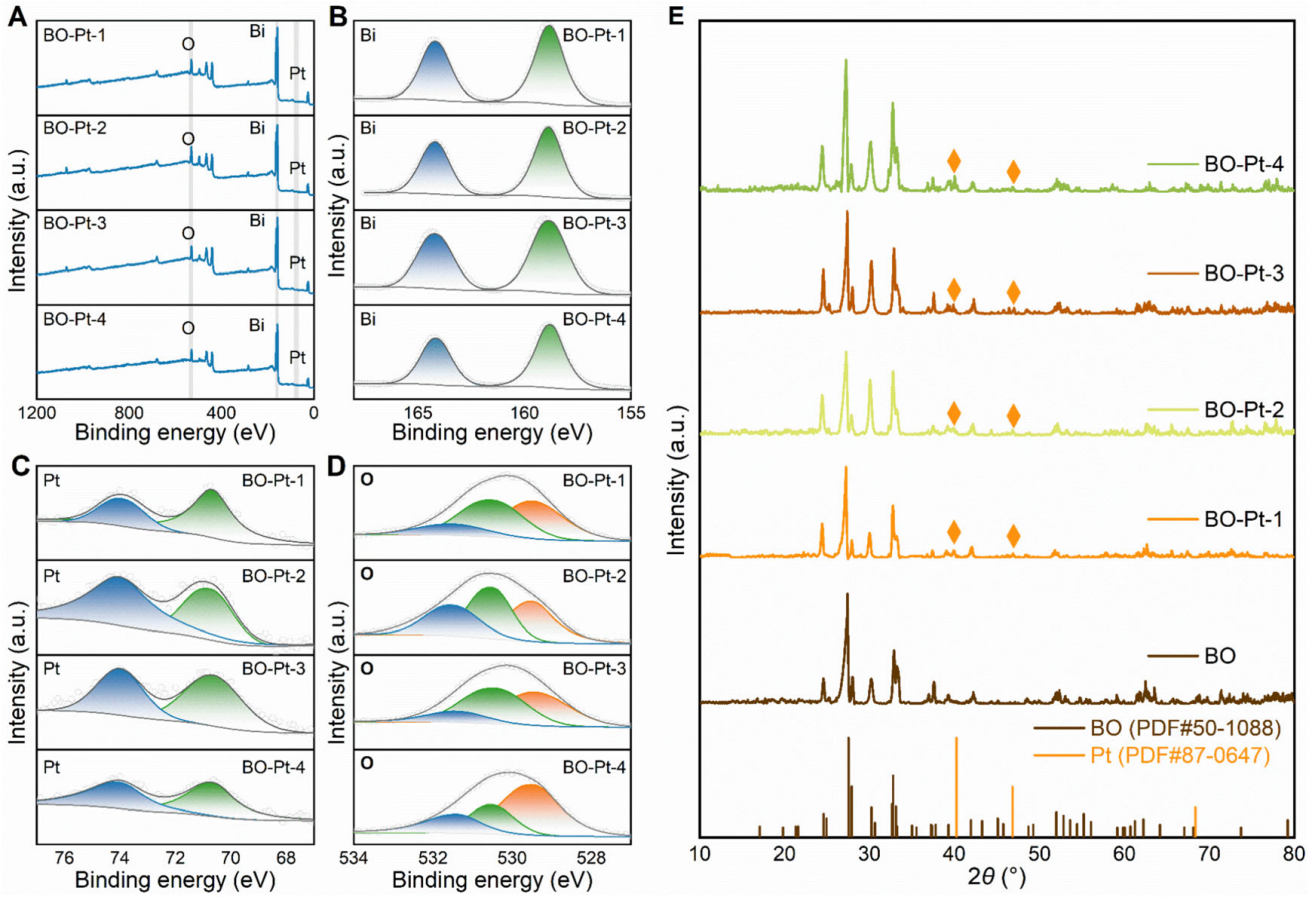
**A** XPS spectra of the Bi_2_O_3_ and the Bi_2_O_3_-Pt nanocomposite; **B**–**D** XPS spectra of Bi_2_O_3_ and Bi_2_O_3_-Pt nanocomposite for the Bi 4f, Pt 4f and O 1s; **E** XRD patterns of the Bi_2_O_3_ and the Bi_2_O_3_-Pt-1/2/3/4 nanocomposite

The XRD patterns of Bi_2_O_3_-Pt-0/1/2/3/4 (Fig. 2E) contain intense diffraction peaks consistent with the standard pattern of Bi_2_O_3_ (JCPDS: 50–1088) [6]. Three additional diffraction peaks appear at 40.246°, 46.814°, and 68.361° in the patterns of the Bi_2_O_3_-Pt samples, corresponding to the (111), (200), and (220) planes of Pt (JCPDS: 87–0647) [19], indicating that Pt was successfully decorated onto the surface of Bi_2_O_3_. These results are consistent with the HR-TEM observations.

The chemical composition and states of the Bi_2_O_3_-Pt-1/2/3/4 nanocomposites were analyzed using XPS. As depicted in Fig. 2A, the XPS spectra confirmed the presence of Bi, O, and Pt in the nanocomposites, with highly consistent peak profiles across all four samples. Figure 2B indicates that the Bi 4f spectra exhibit two prominent peaks corresponding to Bi 4f_7/2_ and Bi 4f_5/2_ [20, 21]. These peaks appear at binding energies of 158.5 and 163.8 eV, respectively, which are characteristic of the Bi^3+^ oxidation state. The Pt 4f_7/2_ and 4f_5/2_ peaks (Fig. 2C) are deconvoluted into those corresponding to metallic Pt^0^ species, located at binding energies of 70.65 and 74.0 eV, respectively [22]. Similarly, the O 1s spectra (Fig. 2D) display three asymmetric peaks at 529.5, 530.5, and 531.4 eV, which are attributed to lattice oxygen (O_L_) originating from Bi–O bonds, oxygen vacancies (O_V_), and chemisorbed surface oxygen (O_C_) species, respectively [13].

After fabricating the Pt-decorated Bi_2_O_3_-based gas sensors, their gas-sensing characteristics were evaluated using a WS-30A gas sensing system at 60% RH. All measurements were conducted in a fume hood. Additional experimental details are provided in the Supporting Information. The sensor response (*S*) is defined by the change in resistance and is calculated using Eq. (2):

2 $$\begin{array}{c}S=\frac{{R}_{\text{a}}}{{R}_{\text{g}}}\end{array}$$

where *R*_a_ denotes the resistance of the sensor in ambient air, and *R*_g_ represents its resistance in the presence of the target analyte. To further evaluate sensor performance, the baseline resistance was measured. As illustrated in Fig. S2, the *R*_a_ value of the Bi_2_O_3_-Pt sensors increases substantially with increasing Pt decoration ratio at 150 °C. This can be explained based on the fact that introducing Pt into a semiconductor with a different work function forms a Schottky barrier at the interface, which hinders carrier transport and consequently increases the device resistance.

**Fig. 3:**
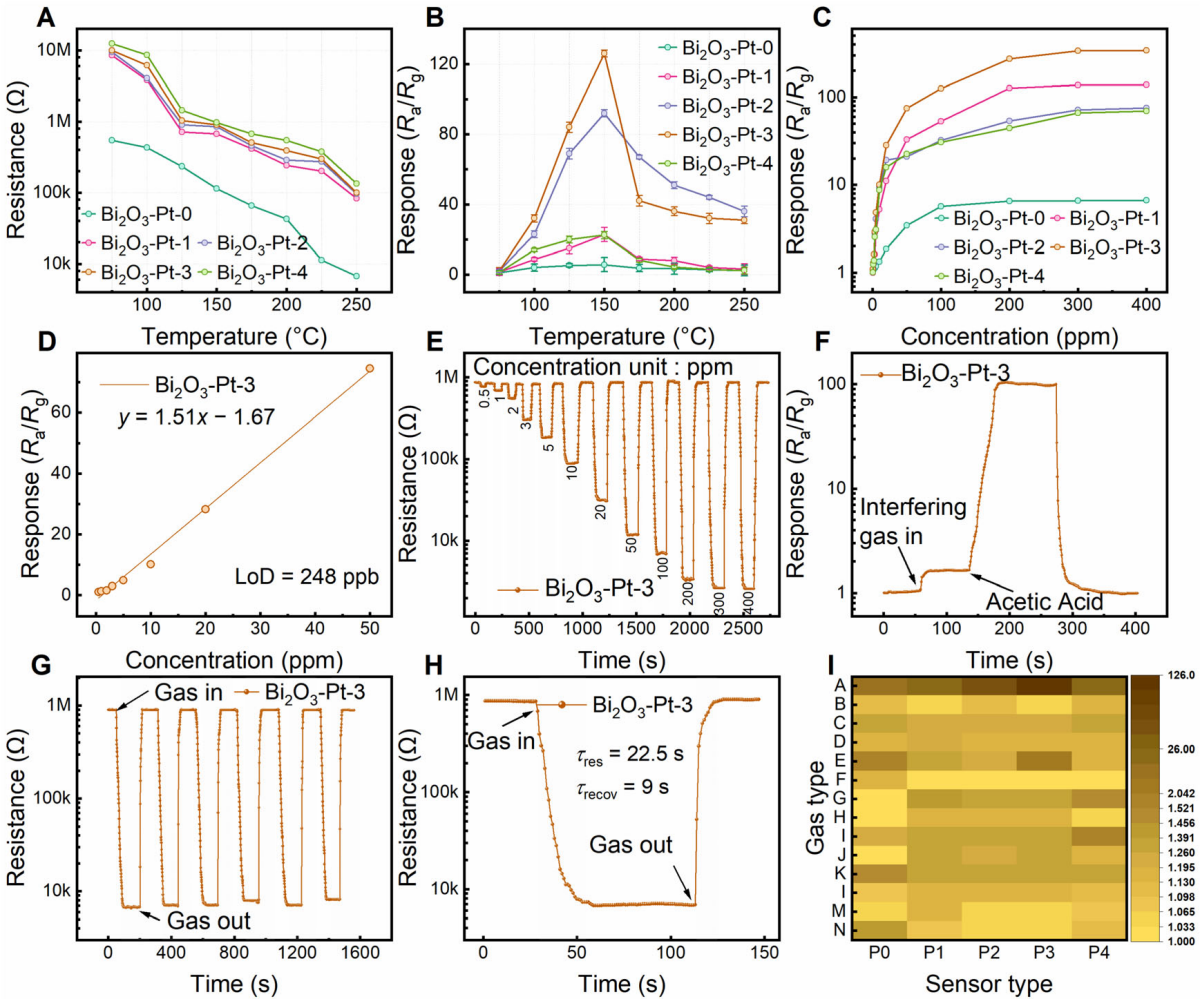
**A** Baseline resistances of Bi_2_O_3_ and Bi_2_O_3_-Pt nanocomposite at varying operating temperatures; **B** responses of Bi_2_O_3_ and Bi_2_O_3_-Pt nanocomposite toward acetic acid (100 ppm) at varying operating temperatures; **C** responses of Bi_2_O_3_ and Bi_2_O_3_-Pt nanocomposite toward to different gas concentrations at their respective working temperature; **D** linear fitting of responses to different acetic acid concentrations (1–50 ppm) for Bi_2_O_3_-Pt-3; **E** dynamic resistance curves of Bi_2_O_3_-Pt-3 when exposed to different gas concentrations; **F** the response of Bi_2_O_3_-Pt-3 to interference from other gases; **G** repeatability of Bi_2_O_3_-Pt-3 to 100 ppm acetic acid at 150 °C; **H** response/recovery times of Bi_2_O_3_-Pt-3 toward 100 ppm acetic acid at 150 °C; **I** responses of Bi_2_O_3_ and Bi_2_O_3_-Pt nanocomposite toward different gases (100 ppm) at 150 °C

It is acknowledged that the semiconductor’s sensor devices highly depend on the operation temperature, due to the inherent characteristics of semiconductors and the mechanism of MOS-based sensors [23], as indicated in Eq. (3). Specifically, changes in temperature (*T*) can substantially influence the carrier concentration (*n*_0_) in the conduction band (*E*_C_), thereby altering the baseline resistance:

3 $$\begin{array}{c}{n}_{0}=2{\left(\frac{{m}_\text{n}^{*}{k}_{0}T}{2\uppi {\hbar }^{2}}\right)}^\frac{3}{2}\text{exp}\left(-\frac{{E}_{\text{C}}-{E}_{\text{F}}}{{k}_{0}T}\right)\end{array}$$

where *m*_n_^*^ denotes the effective mass of the electron, *k*_0_ represents Boltzmann’s constant, and $\hbar$ denotes the reduced Plank’s constant ($\hbar$ = *h*2$\uppi$^−1^). *E*_F_ represents the Fermi energy. Therefore, the concentration of conduction electrons in semiconductor materials is influenced by both temperature and the presence of surface-modifying elements. As illustrated in Fig. 3A, baseline resistance decreases with increasing temperature.

The sensor response is strongly influenced by operating temperature. This effect arises because both the carrier concentration and the activity of gas molecules are temperature dependent. Figure 3B reveals that the Bi_2_O_3_-Pt-0/1/2/3/4 sensors exhibit their highest response to acetic acid at 150 °C. Furthermore, as the temperature rises, the sensor device’s response to acetic acid within the temperature range (100 to 250 °C) exhibits an initial increase followed by a subsequent decrease. At lower temperatures, electron migration within the MOS is limited, and the adsorbed acetic acid molecules do not have sufficient energy to fully react with surface-adsorbed oxygen, resulting in a low sensor response. However, as the temperature rises, two factors contribute to an enhanced response: First, acetic acid molecules gain sufficient energy to participate in redox reactions [24]. Second, the elevated temperature promotes the conversion of lattice oxygen into adsorbed oxygen, which subsequently reacts with acetic acid [25]. However, at even higher temperatures, sensor response diminishes owing to the reduced adsorption of acetic acid molecules onto the sensor surface [26]. The sensor response reaches a maximum at a Pt loading of 3 wt%, following which it declines, likely owing to changes in surface catalytic activity. This is because increasing Pt content initially enhances the number of active sites, thereby promoting surface reactivity. However, excessive Pt functionalization may reduce the effective activation area of the gas-sensitive material despite the increased presence of the catalytic metal [27].

Figure 3C reveals that the sensor response improves with increasing acetic acid concentration, eventually saturating around 300 ppm. Next, using linear fitting, the detection limits (LoDs) of the Bi_2_O_3_-Pt-0/1/2/3/4 sensors were estimated, with Bi_2_O_3_-Pt-3 achieving a low LoD of 248 ppb, as depicted in Figs. 3D, S3. Figures 3E, S4 illustrate the dynamic response and recovery behaviors of the Bi_2_O_3_-Pt-0 and Bi_2_O_3_-Pt-3 sensors to acetic acid in the range of 1–400 ppm at 150 °C. These results indicate that Pt decoration lowers the LoD and enhances the overall sensor response.

Because gas sensors typically operate in mixed-gas environments, evaluating their performance under complex gaseous atmospheric conditions is essential. Accordingly, response measurements for the Bi_2_O_3_-Pt-3 sensor were conducted in a complex gas environment containing acetic acid (A) and a range of potential interfering gases, including triethylamine (B), acetone (C), tetrahydrofuran (D), methanol (E), ethylene glycol (F), isopropanol (G), benzene (H), ethanol (I), acetonitrile (J), dimethylformamide (K), formaldehyde (L), ammonia (M), and dichloromethane (N). As depicted in Fig. 3F, the Bi_2_O_3_-Pt-3 sensor exhibits negligible response to the interfering gases. Therefore, Bi_2_O_3_-Pt-3 demonstrates excellent selectivity toward acetic acid in the presence of interfering species. RH is well-known to adversely affect the sensing performance of gas sensors. Figure S5 illustrates the effect of RH on the Bi_2_O_3_-Pt-3 sensor at an acetic acid concentration of 100 ppm. At 90% RH, the sensor response decreases by 32.5%. This reduction is primarily attributed to competition between water and acetic acid molecules for surface oxygen vacancy consumption, which inhibits the catalytic oxidation of acetic acid [28].

Sensor stability is crucial for practical applications. As depicted in Figs. 3G, S6, the Bi_2_O_3_-Pt-0/1/2/3/4 sensors display excellent repeatability after five cycles of exposure to 100 ppm acetic acid at their respective operating temperatures. Furthermore, the sensors maintained stable performance after one month, as illustrated in Fig. S7.

Response time is a critical parameter for evaluating the practical efficiency of gas sensors. Accordingly, the response and recovery times of Bi_2_O_3_-Pt-0/1/2/3/4 sensors exposed to 100 ppm acetic acid were measured at their respective operating temperatures, as depicted in Figs. 3H, S8. The results indicate that Pt decoration optimizes the reaction time of the sensing material.

Ensuring the sensors' ability to identify target gases in complex gas environments is critical for practical applications. To evaluate the sensors’ selectivity, their responses to various gases (A–N, each at 100 ppm) were measured at 150 °C. As depicted in Figs. 3I, S10, the Bi_2_O_3_-Pt-0/1/2/3/4 sensors exhibit a notably higher response to acetic acid than to other gases, confirming their good selectivity.

To benchmark performance, the sensing characteristics of the Bi_2_O_3_-Pt-3 sensor were compared with those of other acetic acid gas sensors, as depicted in Fig. S9 and Table S2. Compared to the other materials, the Bi_2_O_3_-Pt-3 nanomaterial displayed excellent performance.

The gas sensing mechanism relies on changes in device resistance governed by interactions between target gases and chemisorbed oxygen species. Oxygen molecules adsorb onto the surface of the sensing material and capture electrons to form chemisorbed oxygen species (O_2_, O^−^, O_2_^−^) [29–31]. These reactions are summarized in Eqs. (4–7).

4 $$\begin{array}{c}{\text{O}}_{2} \left(\text{gas}\right) \to {\text{O}}_{2} \left(\text{ads}\right)\end{array}$$

5 $$\begin{array}{c}{\text{O}}_{2} \left(\text{ads}\right)+{\text{ e}}^{-} \to {\text{O}}_{2}^{-} \left(\text{ads}\right)\end{array}$$

6 $$\begin{array}{c}{\text{O}}_{2}^{-} \left(\text{ads}\right)+{\text{ e}}^{-} \to 2{\text{O}}^{-} \left(\text{ads}\right)\end{array}$$

7 $$\begin{array}{c}2{\text{O}}^{-} \left(\text{ads}\right) + {\text{e}}^{-} \to {\text{O}}^{2-} \left(\text{ads}\right)\end{array}$$

**Fig. 4:**
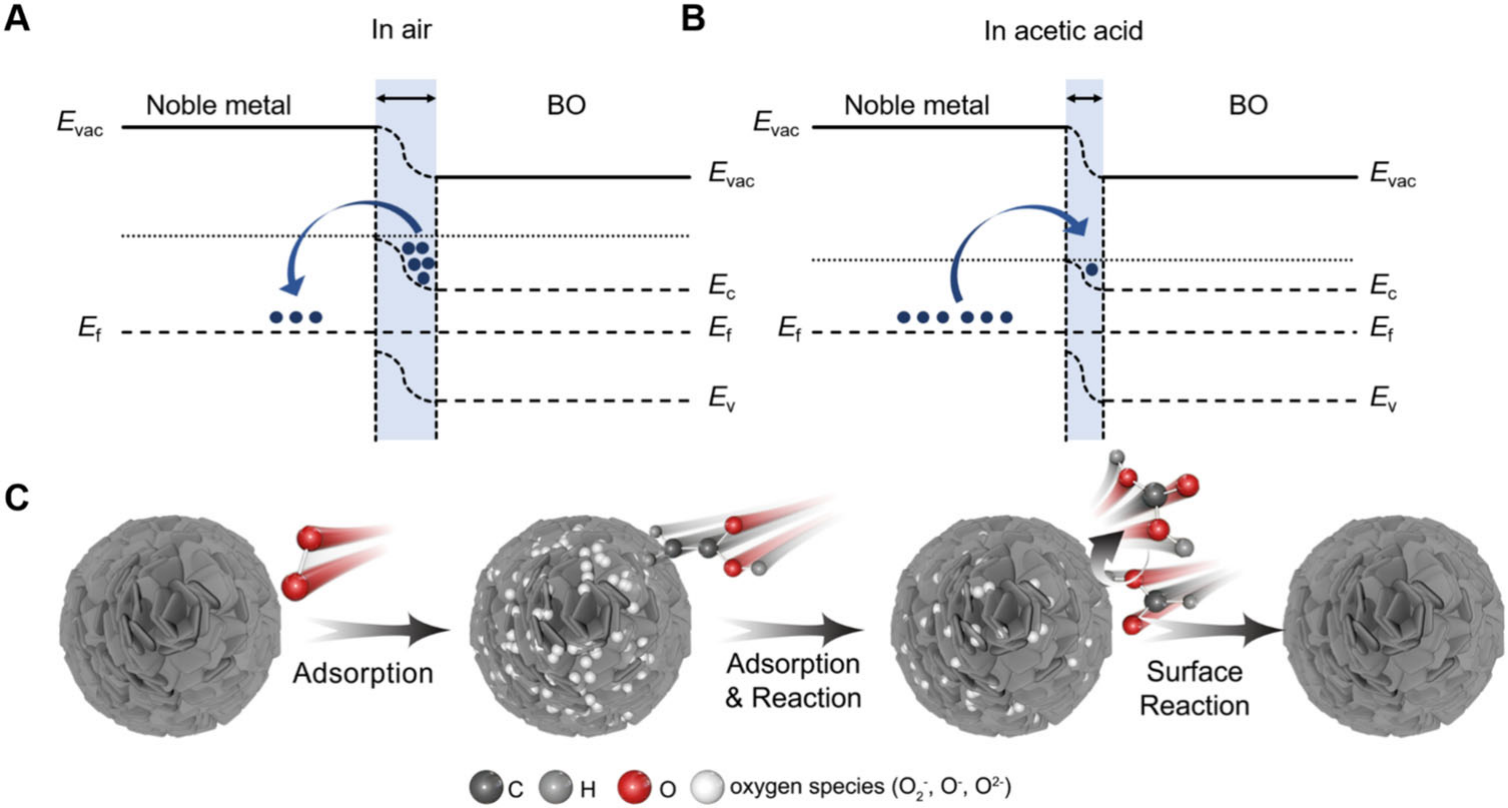
**A**, **B** Schematic energy band diagram of noble metal decorated Bi_2_O_3_ in air and acetic acid. **C** Schematic illustration of the chemical sensitization of the Pt-decorated Bi_2_O_3_ in air and acetic acid

When exposed to acetic acid, the oxygen species react with the gas molecules, releasing electrons back into the conduction band (*E*_C_). This increases electron concentration in *E*_C_, leading to a decrease in resistance. As reported in our previous paper [13], the redox reaction of acetic acid proceeds in stages (Fig. 4C). First, acetic acid (CH_3_COOH) undergoes a decarboxylation reaction with surface oxygen species (O_2_, O^−^, O_2_^−^) to form formic acid (HCOOH). Next, formic acid reacts with additional oxygen species to produce carbonic acid (H_2_CO_3_). Finally, H_2_CO_3_ desorbs from the surface, releasing CO_2_ and H_2_O, as described by Eqs. (8–10).

8 $$\begin{array}{*{20}c} {C{\text{H}}_{3} {\text{COOH}} + 2{\text{O}}^{ - } \to 2{\text{HCOOH}} + 2{\text{e}}^{ - } } \\ \end{array}$$

9 $$\begin{array}{*{20}c} {{\text{HCOOH}} + {\text{O}}^{ - } \to {\text{ H}}_{2} {\text{CO}}_{3} + {\text{e}}^{ - } } \\ \end{array}$$

10 $$\begin{array}{*{20}c} {{\text{H}}_{2} {\text{CO}}_{3} \to {\text{CO}}_{2} + {\text{H}}_{2} {\text{O}}} \\ \end{array}$$

The considerable enhancement in gas sensitivity following Pt decoration can be attributed to two primary factors: electron sensitization and catalytic activation. In the electron sensitization process, the mismatch in work functions between Pt and Bi_2_O_3_ drives electron transfer from Bi_2_O_3_ to Pt, leading to the formation of a potential barrier. As illustrated in Fig. 4A, B, the higher work function of Pt causes electrons to spontaneously migrate from the conduction band of Bi_2_O_3_ to Pt until their Fermi levels equilibrate at the interface [32]. This charge transfer promotes the formation of a Schottky barrier and increases the thickness of the electron depletion layer. These effects suppress electron–hole recombination and induce pronounced resistance changes upon gas exposure, substantially enhancing the sensor response [33]. Additionally, the catalytic action of Pt NPs enables the dissociation of oxygen molecules, producing additional chemisorbed oxygen species. These oxygen species are preferentially adsorbed and dissociated on Pt into reactive O^−^ species, which react with acetic acid to release electrons, causing a rapid decrease in resistance and markedly improving sensing performance.

## Conclusion

In this study, Pt-decorated Bi_2_O_3_ microspheres were synthesized through nanostructure engineering and surface modification using the noble metal Pt. SEM and TEM analyses confirmed that the surfaces of the permeable Bi_2_O_3_ microspheres were decorated with Pt nanoparticles. Among all samples, Bi_2_O_3_-Pt-3 exhibited the highest response (126) to 100 ppm acetic acid, along with rapid response/recovery times (22.5/9 s, respectively), strong anti-interference capability, and high selectivity at a relatively low operating temperature of 150 °C. This enhanced sensing performance of Bi_2_O_3_-Pt-3 is attributed not only to the high permeability of Bi_2_O_3_ but also to the synergistic catalytic effect of Pt nanoparticles. Overall, this study offers a promising strategy for synthesizing Pt-decorated Bi_2_O_3_ microspheres derived from the permeable precursor Bi_2_O_2_CO_3_, which shows strong potential for application in high-performance gas sensors.

## Supplementary Information

Below is the link to the electronic supplementary material.

Supplementary file1 (DOCX 5938 KB)

## Data Availability

The data that support the findings of this study are available from the corresponding author upon reasonable request.
