# Supplementary material for: Structural design of highly permeable Bi2O3 microspheres decorated by Pt-nanoparticles: facile synthesis and acetic acid sensing performance
Source: Rare Metals. 2025 Jul 10;44(9):6417–25. doi: 10.1007/s12598-025-03391-y (PMC12339603; doi:10.1007/s12598-025-03391-y)
Supplement: Supplementary file 1 — Supplementary file1 (DOCX 5938 KB) [file 12598_2025_3391_MOESM1_ESM.docx]

Supporting Information

Structural design of highly permeable Bi_2_O_3_ microspheres decorated by Pt-nanoparticles: facile synthesis and acetic acid sensing performance

**Fan Yang ^1^, Junning Zhang ^1^, Chao Zhang ^1^, Xinda Xu ^1^, Bing Li ^3^*, Woochul Yang ^2^*, Wanfeng Xie ^1,2^***

*^1^* *College of Electronics and Information, Qingdao University, Qingdao 266071, China*

*^2^ Department of Physics, Dongguk University, Seoul 04620, Republic of Korea*

*^3^ Institute for Materials Discovery, Department of Chemistry, University College London, London, WC1E 7JE UK*

* To whom correspondence should be addressed

Prof. Wanfeng Xie, Email: [wfxie@qdu.edu.cn](mailto:wfxie@qdu.edu.cn)

Prof. Woochul Yang, [wyang@dongguk.edu](mailto:wyang@dongguk.edu)

Prof. Bing Li, [bing.li@ucl.ac.uk](mailto:bing.li@ucl.ac.uk)


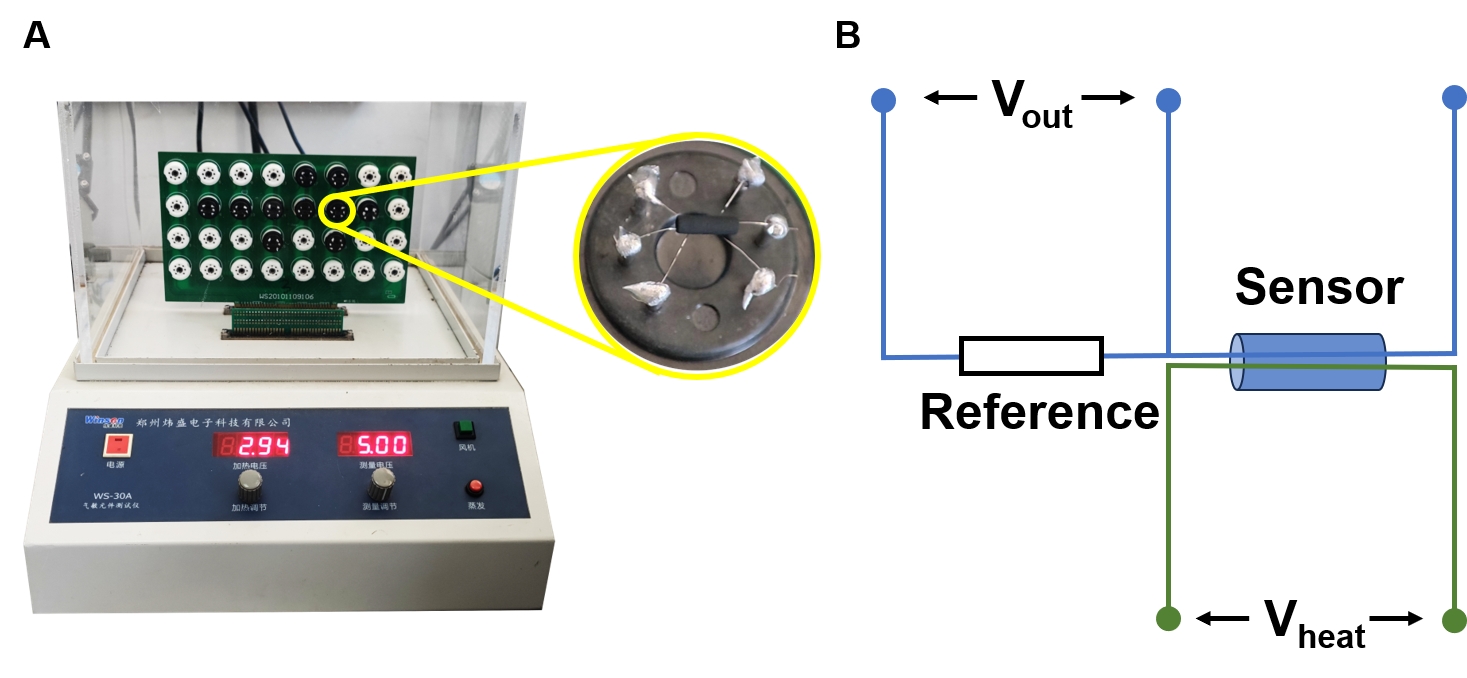


**Fig. S1** (a) Digital photographs of gas-sensing measurement; (b) The inner working circuit of the gas-sensing device.


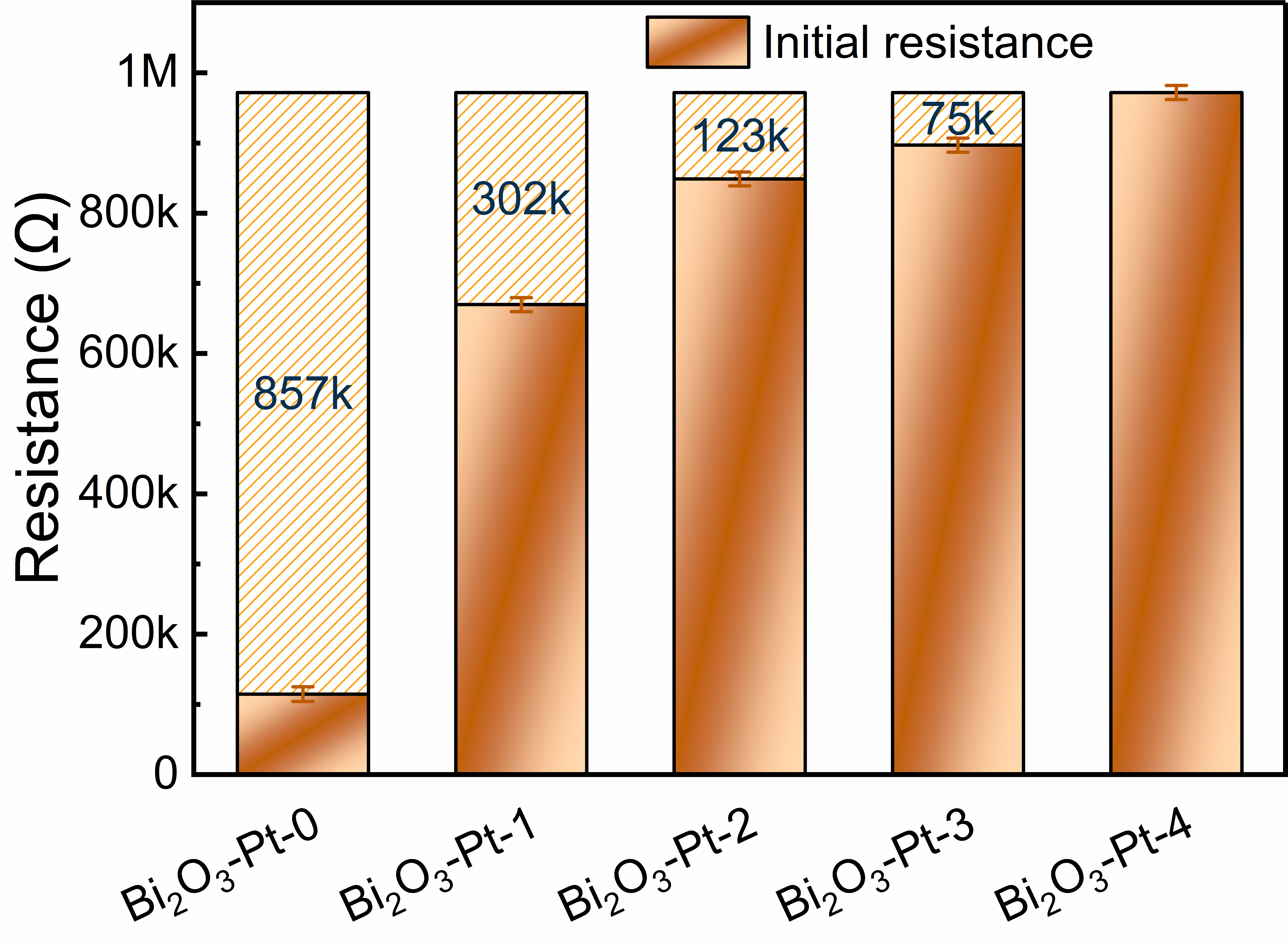


**Fig. S2** Baseline resistances of Bi_2_O_3_-Pt-0/1/2/3/4 at 150 ℃.


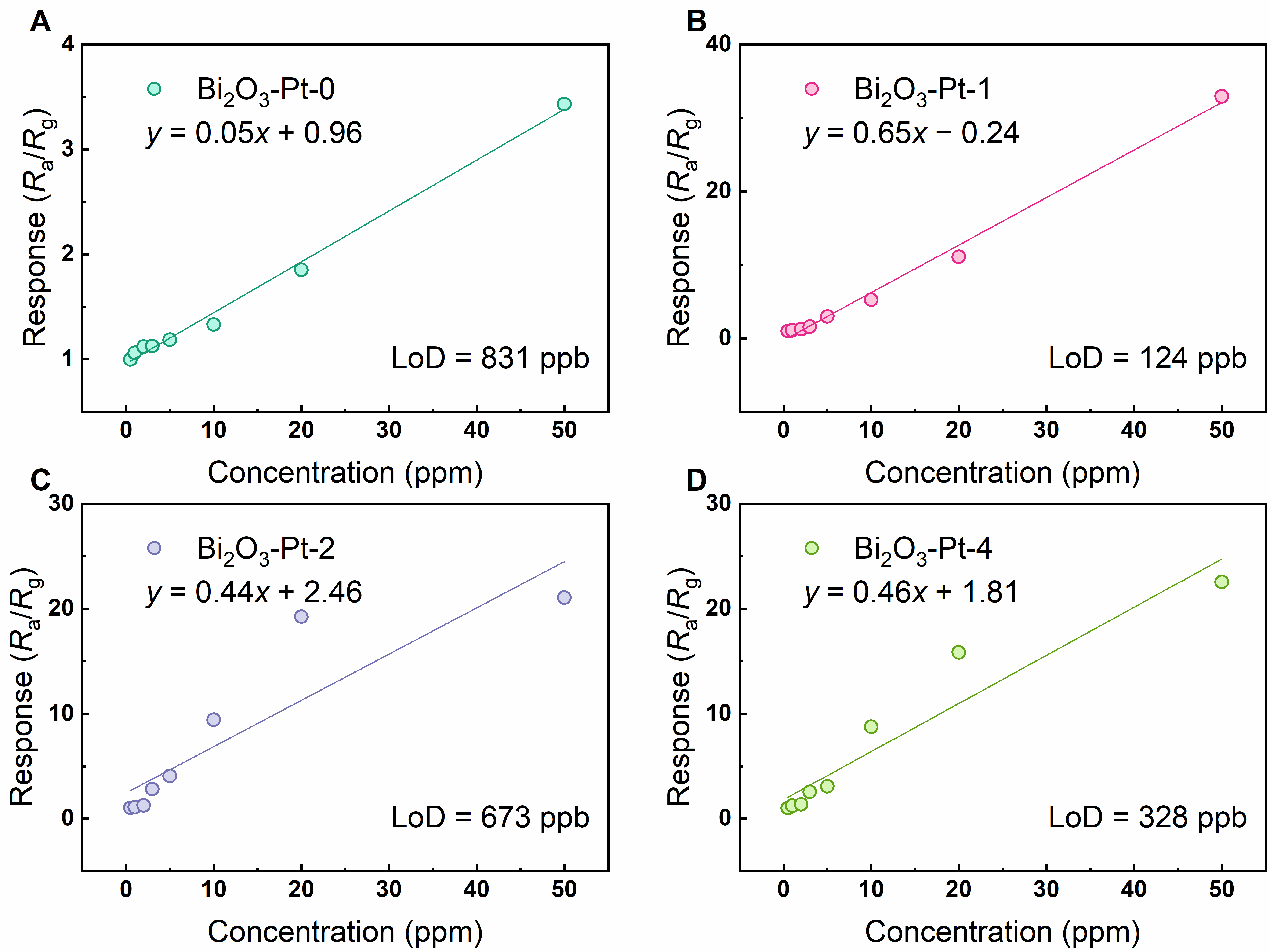


**Fig. S3** The linear fitting to the relationship between response and concentration (1 - 50 ppm) of acetic acid for Bi_2_O_3_-Pt-0/1/2/4 at 150 ℃


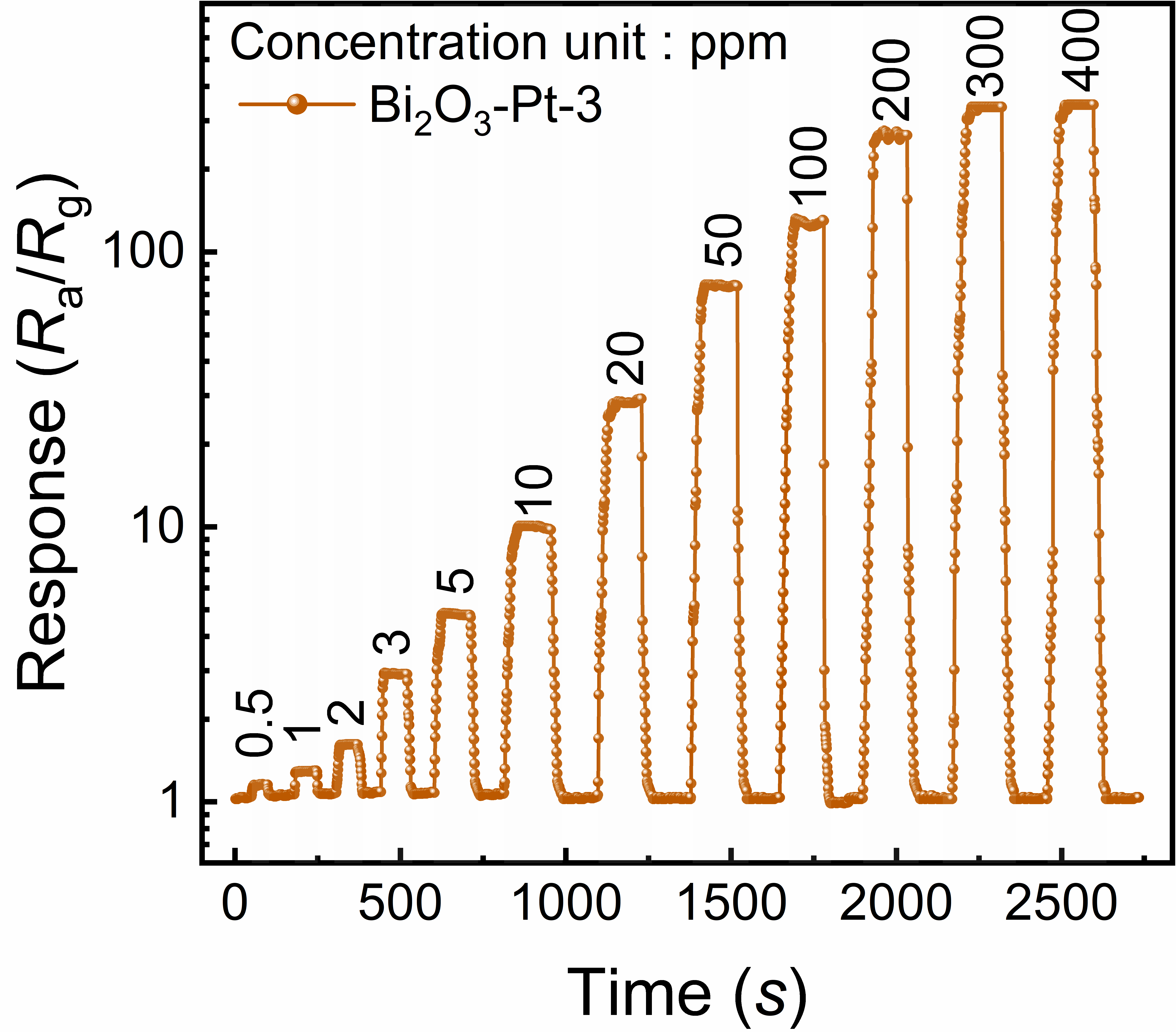


**Fig. S4** Dynamic response changes of Bi_2_O_3_-Pt-3 based sensors when exposed to 1 - 300 ppm acetic acid at 150 ℃.


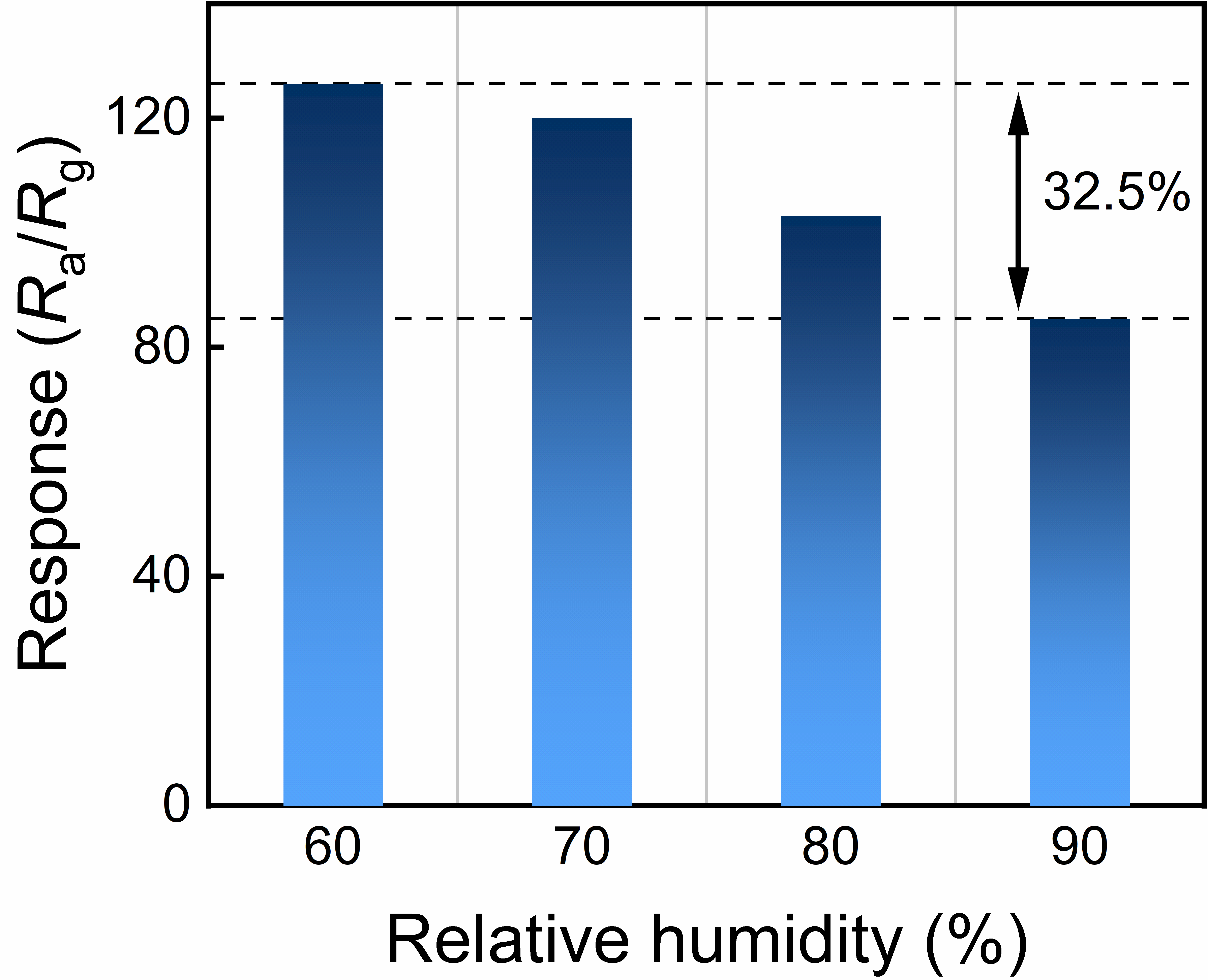


**Fig. S5** The response of Bi_2_O_3_-Pt-3 to 100 ppm acetic acid in different relative humidity conditions at 25 ℃, and the operating temperature of the gas sensor is 150 ℃.


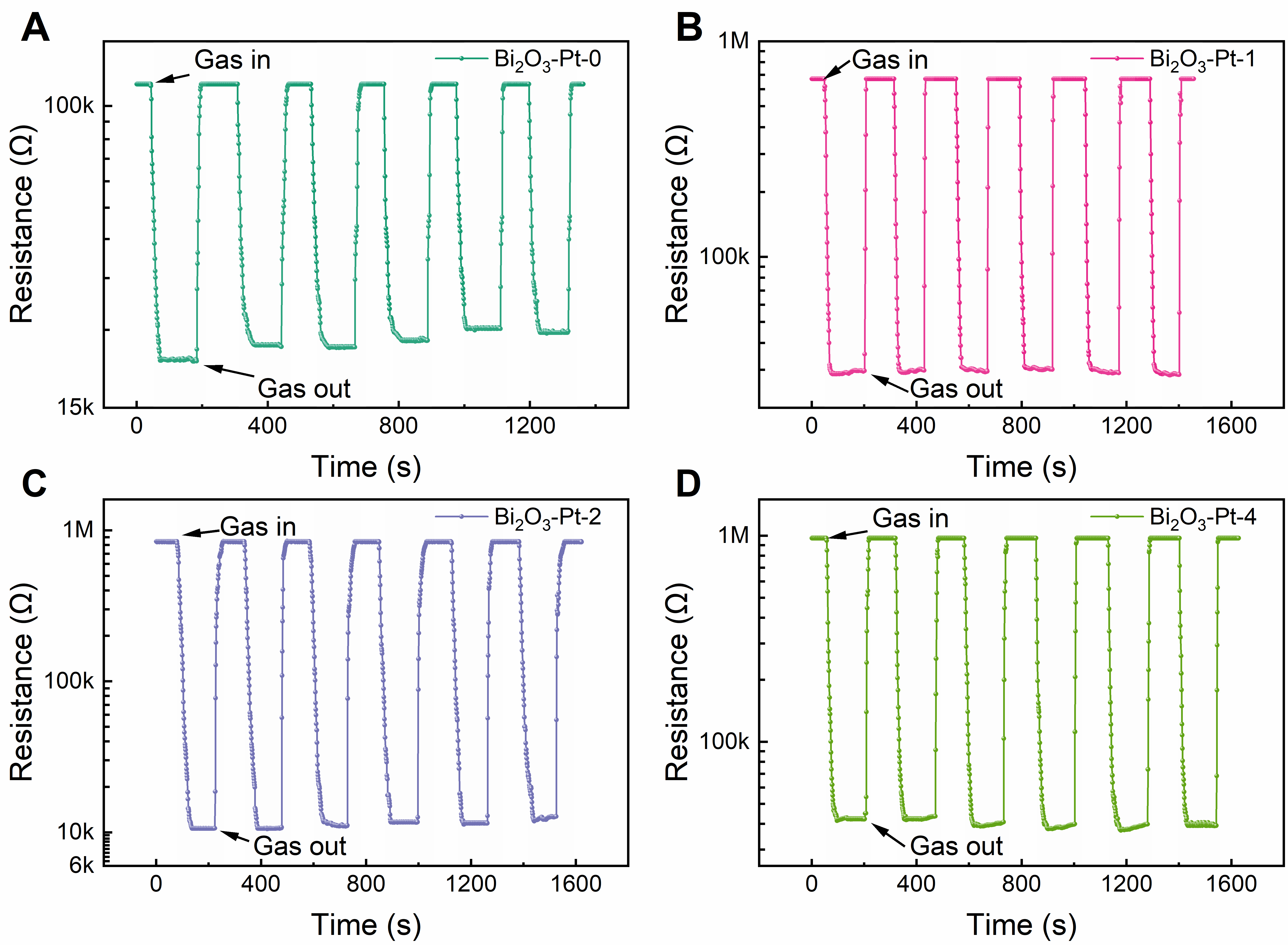


**Fig. S6** Repeatability of Bi_2_O_3_-Pt-0/1/2/4 toward 100 ppm acetic acid at 150 ℃.


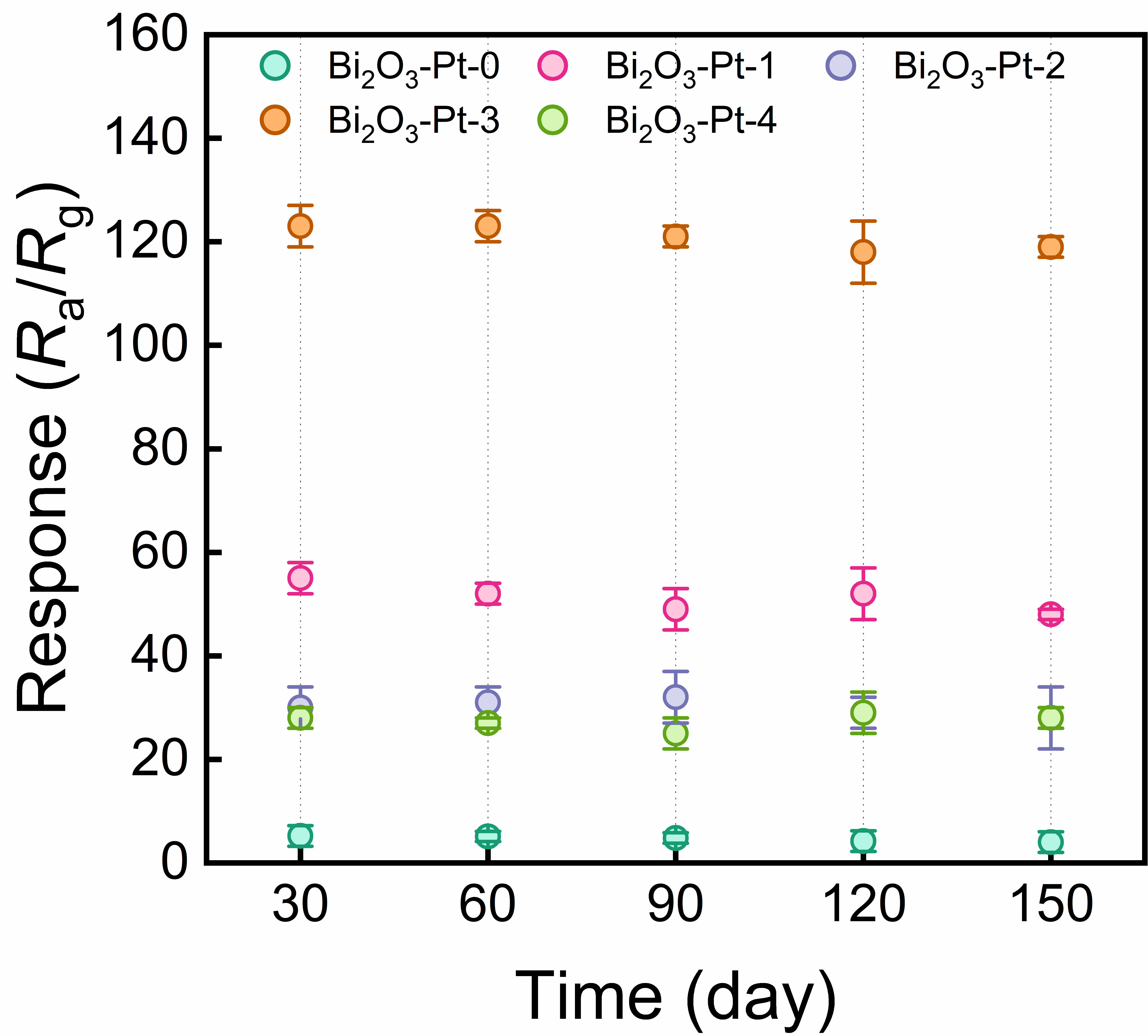


**Fig. S7** Long-term stability of Bi_2_O_3_-Pt-0/1/2/3/4 toward 100 ppm acetic acid at 150 ℃.


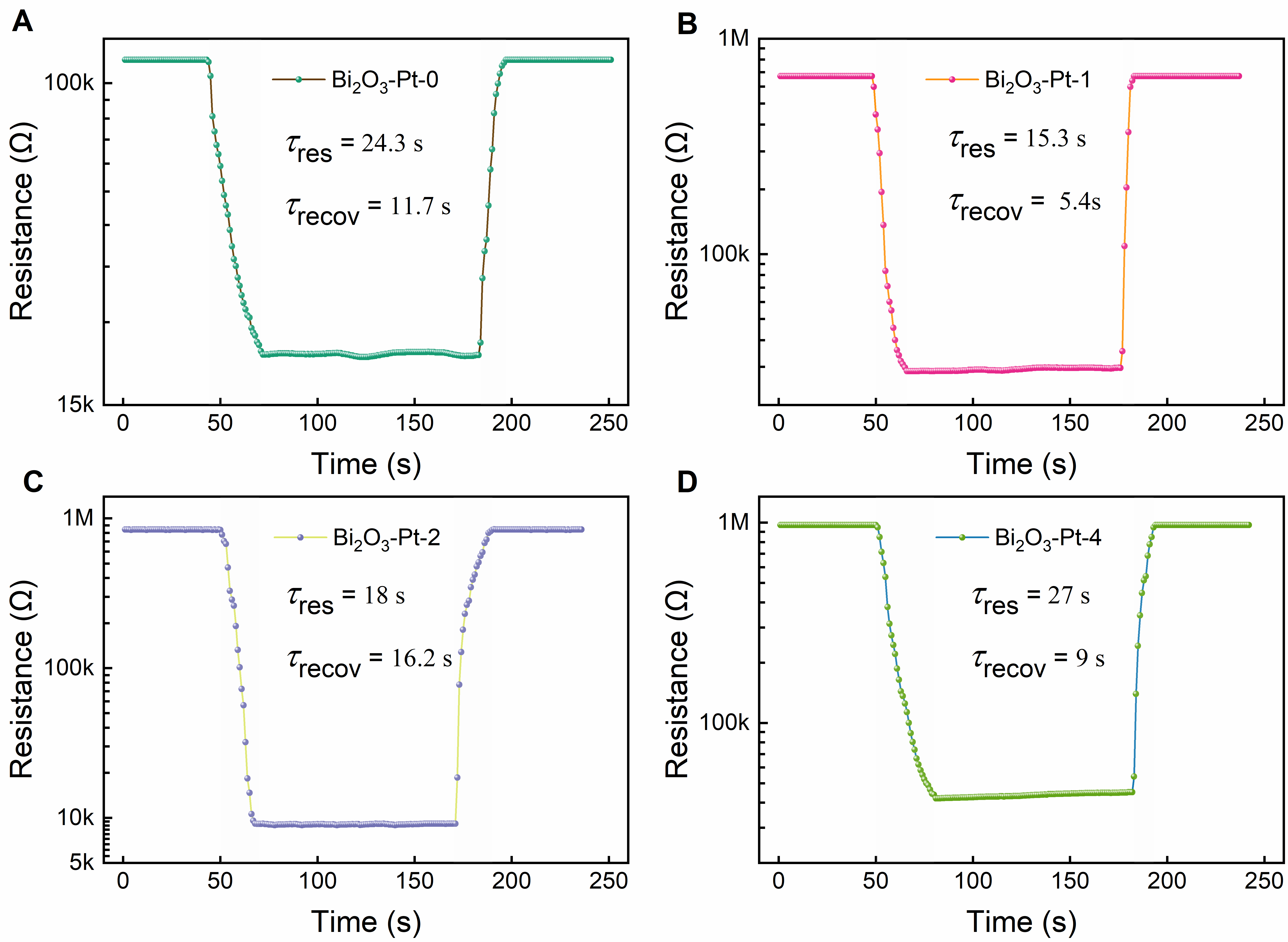


**Fig. S8** Response/recovery times of Bi_2_O_3_-Pt-0/1/2/4 toward 100 ppm acetic acid at 150 ℃.


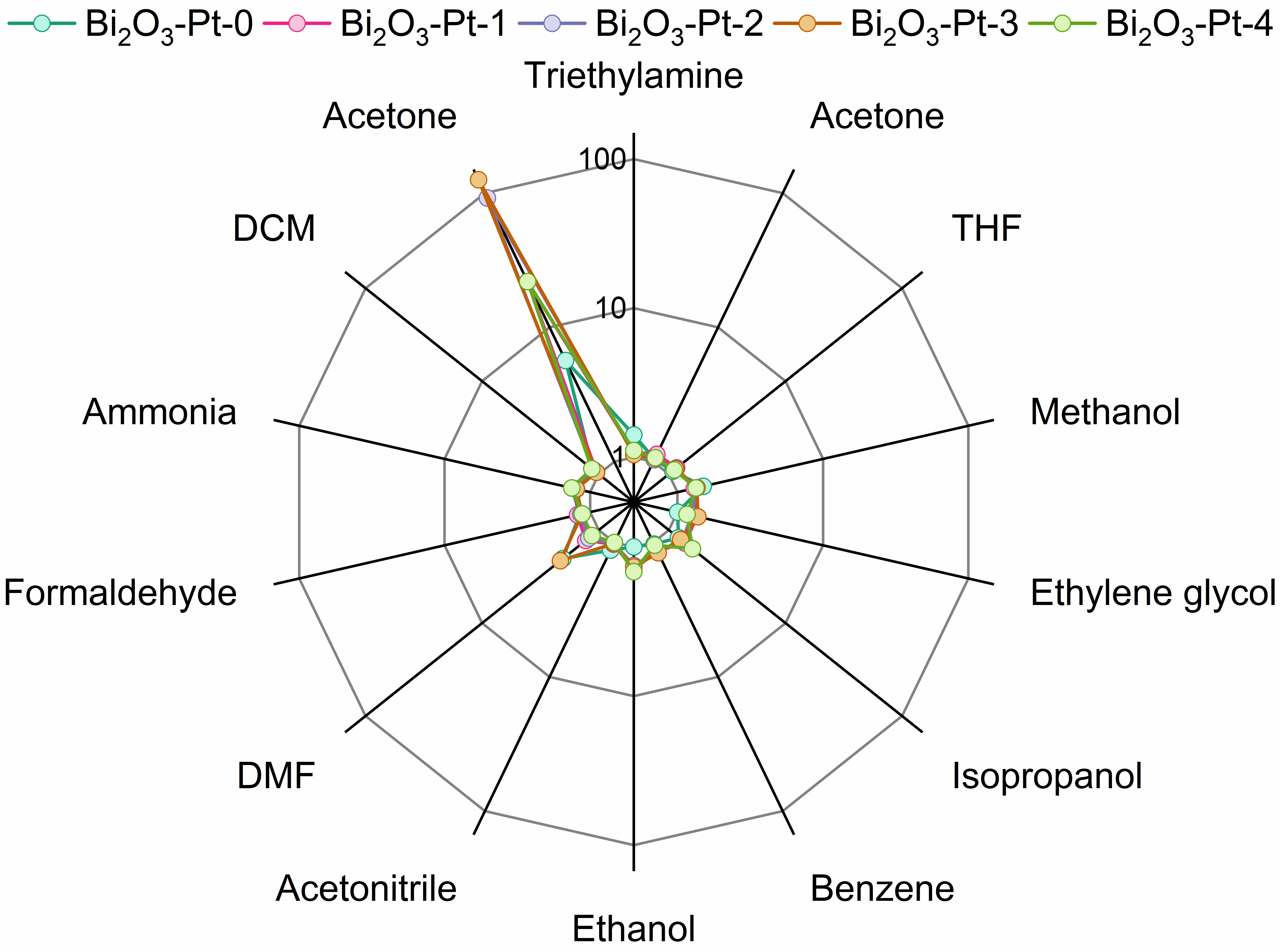


**Fig. S9** Selectivity of Bi_2_O_3_-Pt-0/1/2/3/4 toward 100 ppm of different gases at 150 ℃.


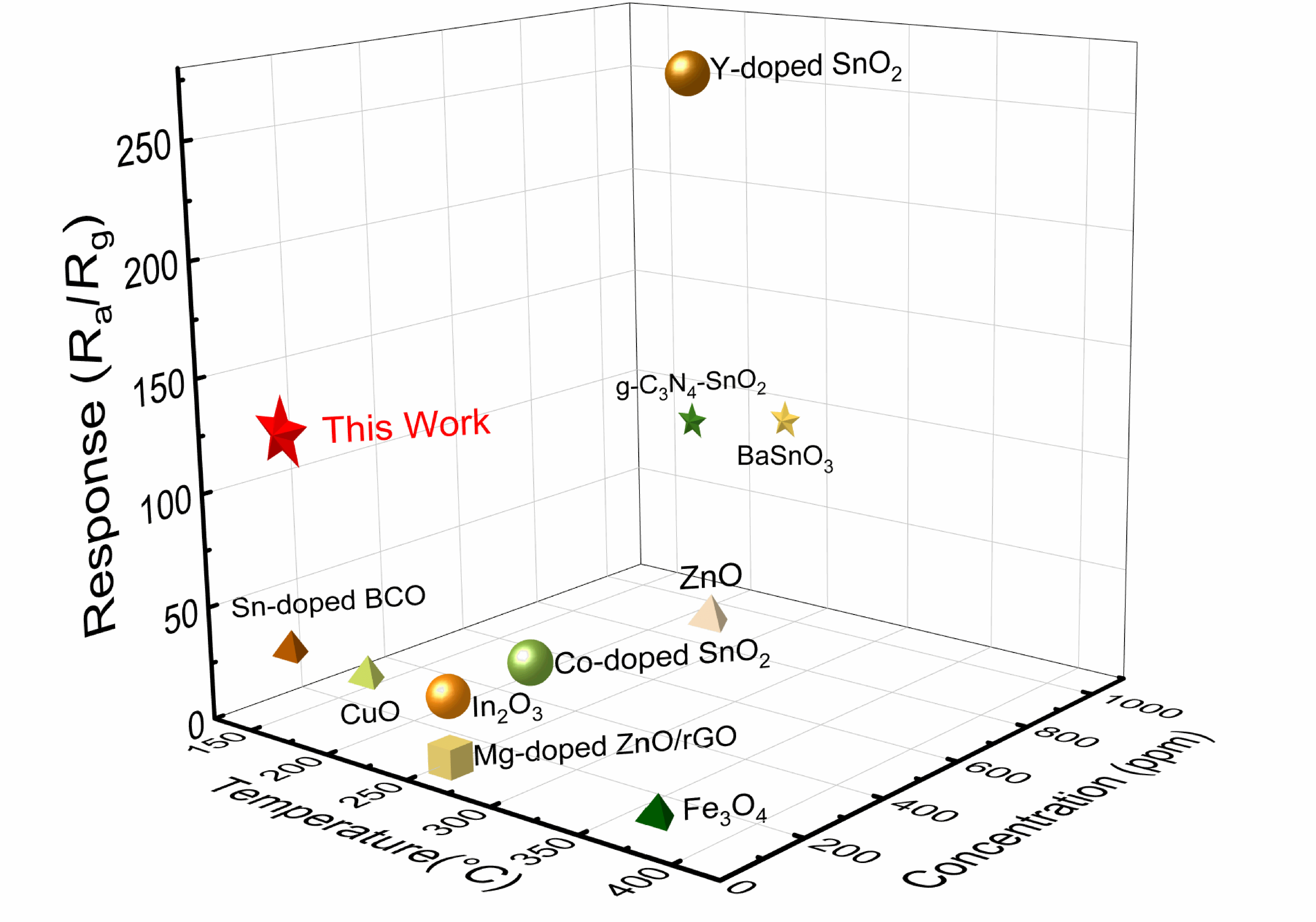


**Fig. S10** The comparison of the sensing performance of acetic acid sensors reported in recent years includes the optimal operating temperature, detected gas concentration, and gas response value.

**Table S1** The Analytical Reagent (AR) of the liquid target gases (%)

| **Target liquid** | **AR (%)** |
| --- | --- |
| Triethylamine | 99.5 |
| Acetone | 99.5 |
| THF | 99.5 |
| Methanol | 99.5 |
| Ethylene glycol | 99.9 |
| Isopropanol | 99.7 |
| Benzene | 99.5 |
| Ethanol | 99.7 |
| Acetonitrile | 99.9 |
| DMF | 99.9 |
| Formaldehyde | 37-40 |
| Ammonia | 25-28 |
| DCM | 99.5 |
| Acetic Acid | 99.5 |

**Table S2** The comparison of the sensing performance of acetic acid sensors reported in recent years includes the optimal operating temperature (temp.), detected gas concentration (conc.), gas response (S), the response/recovery time and Detection limit (Lim).

| **Materials** | **Temp.**  **(℃)** | **Conc.**  **(ppm)** | **S**  **(R_a_/R_g_)** | **τ_res_/τ_recov_**  **(s)** | **Lim**  **(ppm)** | **Ref.** |
| --- | --- | --- | --- | --- | --- | --- |
| Sn-doped BCO | 150 | 100 | 28.8 | 10/44 | 200 | [1] |
| Y-doped SnO_2_ | 300 | 500 | 275.6 | 7/10 | 500 | [2] |
| BaSnO_3_ microtubes | 245 | 1000 | 96.8 | 7/22 | 500 | [3] |
| Mesoporous CuO | 200 | 100 | 27.2 | 71/64 | 100 | [4] |
| Co-doped SnO_2_ nanosheets | 300 | 100 | 52.94 | 12/14 | 200 | [5] |
| g–C_3_N_4_–SnO_2_ composites | 185 | 1000 | 87.7 | 94/160 | 1000 | [6] |
| Fe_3_O_4_/C nanocomposites | 370 | 100 | 4.7 | 6/13 | 1000 | [7] |
| MGO/G composites | RT | 100 | 363 | 50/35 | 100 | [8] |
| ZnO foam | 400 | 100 | 90.37 | 12/13 | 100 | [9] |
| Mg-doped ZnO/rGO | 250 | 100 | 2 | 66/35 | 200 | [10] |
| Pt-decorated BO | 150 | 100 | 126 | 22.5/9 | 200 | This work |

**References**

[1] X.Y. Huang, K. Chen, W. Xie, Y. Li, F. Yang, Y. Deng, et al., Chemiresistive gas sensors based on highly permeable Sn‐doped bismuth subcarbonate microspheres: facile synthesis, sensing performance, and mechanism study, Advanced Functional Materials, 2023;33(45):2304718, https://doi.org/10.1002/adfm.202304718.

[2] L. Cheng, S.Y. Ma, T.T. Wang, J. Luo, X.B. Li, W.Q. Li, et al., Highly sensitive acetic acid gas sensor based on coral-like and Y-doped SnO_2_ nanoparticles prepared by electrospinning, Materials Letters, 2014;137:265-8, https://doi.org/10.1016/j.matlet.2014.09.040.

[3] X.F. Chu, Z.Q. Gan, L.S. Bai, Y.P. Dong, M.N. Rumyantseva, The acetic acid vapor sensing properties of BaSnO microtubes prepared by electrospinning method, Materials Science and Engineering B-Advanced Functional Solid-State Materials, 2020;259:114606, https://doi.org/10.1016/j.mseb.2020.114606.

[4] W.C. Geng, Z.Y. Ma, J.H. Yang, L.B. Duan, F. Li, Q.Y. Zhang, Pore size dependent acetic acid gas sensing performance of mesoporous CuO, Sensors and Actuators B-Chemical, 2021;334:129639, https://doi.org/10.1016/j.snb.2021.129639.

[5] W.J. Bi, S.T. Liu, Preparation of a hierarchical 3D structure composed of Co-doped SnO_2_ nanosheets with excellent gas sensitivity to acetic acid, Materials Science and Engineering B-Advanced Functional Solid-State Materials, 2022;286:116006, https://doi.org/10.1016/j.mseb.2022.116006.

[6] Y. Zhang, J.S. Liu, X.F. Chu, S.M. Liang, L.B. Kong, Preparation of g-C_3_N_4_-SnO_2_ composites for application as acetic acid sensor, Journal of Alloys and Compounds, 2020;832:153355, https://doi.org/10.1016/j.jallcom.2019.153355.

[7] X. Liang, Y. Qin, W. Xie, Z. Deng, C. Yang, X. Su, Facile synthesis of high-stable and monodisperse Fe_3_O_4_/carbon flake-like nanocomposites and their excellent gas sensing properties, Journal of Alloys and Compounds, 2020;818:152898, https://doi.org/10.1016/j.jallcom.2019.152898.

[8] L. He, C. Gao, L. Yang, K. Zhang, X. Chu, S. Liang, et al., Facile synthesis of MgGa_2_O_4_/graphene composites for room temperature acetic acid gas sensing, Sensors and Actuators B: Chemical, 2020;306:127453, https://doi.org/10.1016/j.snb.2019.127453.

[9] M. Turemis, D. Zappi, M.T. Giardi, G. Basile, A. Ramanaviciene, A. Kapralovs, et al., ZnO/polyaniline composite based photoluminescence sensor for the determination of acetic acid vapor, Talanta, 2020;211:120658, https://doi.org/10.1016/j.talanta.2019.120658.

[10] V. Khorramshahi, J. Karamdel, R. Yousefi, High acetic acid sensing performance of Mg-doped ZnO/rGO nanocomposites, Ceramics International, 2019;45(6):7034-43, https://doi.org/10.1016/j.ceramint.2018.12.205.
